# Association of Internal Medicine Point of Care Ultrasound (POCUS) with Length of Stay, Hospitalization Costs, and Formal Imaging: a Prospective Cohort Study

**DOI:** 10.24908/pocus.v8i2.16791

**Published:** 2023-11-27

**Authors:** David M Tierney, Terry K Rosborough, Lynn M Sipsey, Kai Hanson, Claire S Smith, Lori L Boland, Robert Miner

**Affiliations:** 1 Department of Graduate Medical Education, Abbott Northwestern Hospital Minneapolis, MN USA; 2 Allina Health Care Delivery Research Minneapolis, MN USA

**Keywords:** Internal Medicine, Point of Care Ultrasound (POCUS), Diagnostic imaging, bedside ultrasound

## Abstract

**Background: **Point of care ultrasound (POCUS) use has rapidly expanded among internal medicine (IM) physicians in practice and residency training programs. Many benefits have been established; however, studies demonstrating the impact of POCUS on system metrics are few and mostly limited to the emergency department or intensive care setting. The study objective was to evaluate the impact of inpatient POCUS on patient outcomes and hospitalization metrics. **Methods: **Prospective cohort study of 12,399 consecutive adult admissions to 22 IM teaching attendings, at a quaternary care teaching hospital (7/1/2011-6/30/2015), with or without POCUS available during a given hospitalization. Multivariable regression and propensity score matching (PSM) analyses compared multiple hospital metric outcomes (costs, length of stay, radiology-based imaging, satisfaction, etc.) between the “POCUS available” vs. “POCUS unavailable” groups as well as the “POCUS available” subgroups of “POCUS used” vs. “POCUS not used”. **Results: **Patients in the “POCUS available” vs. “POCUS unavailable” group had lower mean total and per-day hospital costs ($17,474 vs. $21,803, p<0.001; $2,805.88 vs. $3,557.53, p<0.001), lower total and per-day radiology cost ($705.41 vs. $829.12, p<0.001; $163.11 vs. $198.53, p<0.001), fewer total chest X-rays (1.31 vs. 1.55, p=0.01), but more chest CTs (0.22 vs 0.15; p=0.001). Mean length of stay (LOS) was 5.77 days (95% CI = 5.63, 5.91) in the “POCUS available” group vs. 6.08 95% CI (5.66, 6.51) in the “POCUS unavailable” group (p=0.14). Within the “POCUS available” group, cost analysis with a 4:1 PSM (including LOS as a covariate) compared patients receiving POCUS vs. those that could have but did not, and also showed total and per-day cost savings in the “POCUS used” subgroup ($15,082 vs. 15,746; p<0.001 and $2,685 vs. $2,753; p=0.04). **Conclusions: **Availability and selected use of POCUS was associated with a meaningful reduction in total hospitalization cost, radiology cost, and chest X-rays for hospitalized patients.

## Background

Point of care ultrasound (POCUS) is quickly being adopted by internal medicine (IM) physicians in practice and being increasingly taught in the majority of IM residencies due to its impact on procedural safety, diagnostic accuracy and efficiency, provider and patient satisfaction, and use as a teaching adjunct in medical education 1, 2, 3, 4, 5, 6, 7, 8. Despite these benefits, studies demonstrating its impact on system efficiency and cost of care are few, difficult to perform, and mostly in the emergency department (ED) and intensive care unit (ICU) settings 9, 10, 11.

A randomized trial to evaluate the impact of POCUS on clinical and system outcomes is impracticable and potentially unethical within a controlled IM setting as it would require withdrawal of POCUS from well-trained, regular users who rely on the tool for optimal care, and in such an environment clinical equipoise would no longer exist 12. We had a unique natural situation that introduced some aspects of a randomized trial within a single group of POCUS-trained teaching hospitalists. Our study objective was to use this environment to compare cost and other metrics among hospitalized patients cared for with and without POCUS availability.

## Methods

### Setting

This prospective observational cohort study took place within a 670-bed teaching hospital with a long-standing inpatient and outpatient IM residency-based POCUS curriculum since 2010. The study period (7/1/2011-6/30/2015) was chosen due to the natural randomization of POCUS device availability among a single group of hospitalists as described below and to evaluate outcomes during the earlier years of a POCUS program. The study was approved by our external IRB (Schulman IRB; Reference 201306503).

### IM POCUS Curriculum & Infrastructure

All first-year IM residents participated in a 5-day, 40-hour internal POCUS course, and completed a 1-month ultrasound rotation—a 1:1 intensive POCUS experience with a core POCUS faculty. Core hospitalist teaching faculty at that time all participated in a 2-day hands-on POCUS course and most proceeded through a mentored, 5+-day, bedside, hands-on experience. All resident-performed exams were mentored by certified faculty at the bedside until the resident was certified. Certification is by individual body area and requires 1) a minimum exam quantity within each body area sub-area (Appendix 1: IMBUS certification criteria), followed by 2) a bedside assessment for POCUS interpretation and clinical integration competency within the body area being certified 13.

### POCUS Devices and Use

During the initial years of our POCUS program, six IM resident teams carried portable ultrasound devices (SonoSite NanoMaxx and EDGE devices with P21 [1-5mHz] phased-array and L25 [13-6mHz] linear transducers; FUJIFILM SonoSite Inc., Bothell, WA), resulting in the potential use of diagnostic POCUS for general ward patients limited to patients assigned to resident teams (hospitalist attending, PGY1 and PGY2 resident, and medical students). POCUS exams were prospectively tracked on a smartphone-based application that recorded a unique patient identifier, exam time and location, body areas and items evaluated, findings, type of ultrasound device, and faculty mentor 14. POCUS was available to intensivists in the ICU and emergency medicine providers in the ED without restriction; exams performed by these non-IM providers were not included in this study. However, POCUS exams performed by the IM team in the ED (such as during the admission exam) or ICU setting were included in the study. POCUS for procedural guidance was not included in this study. POCUS examinations were not billed.

### Patient Group Assignments

**Figure 1:**
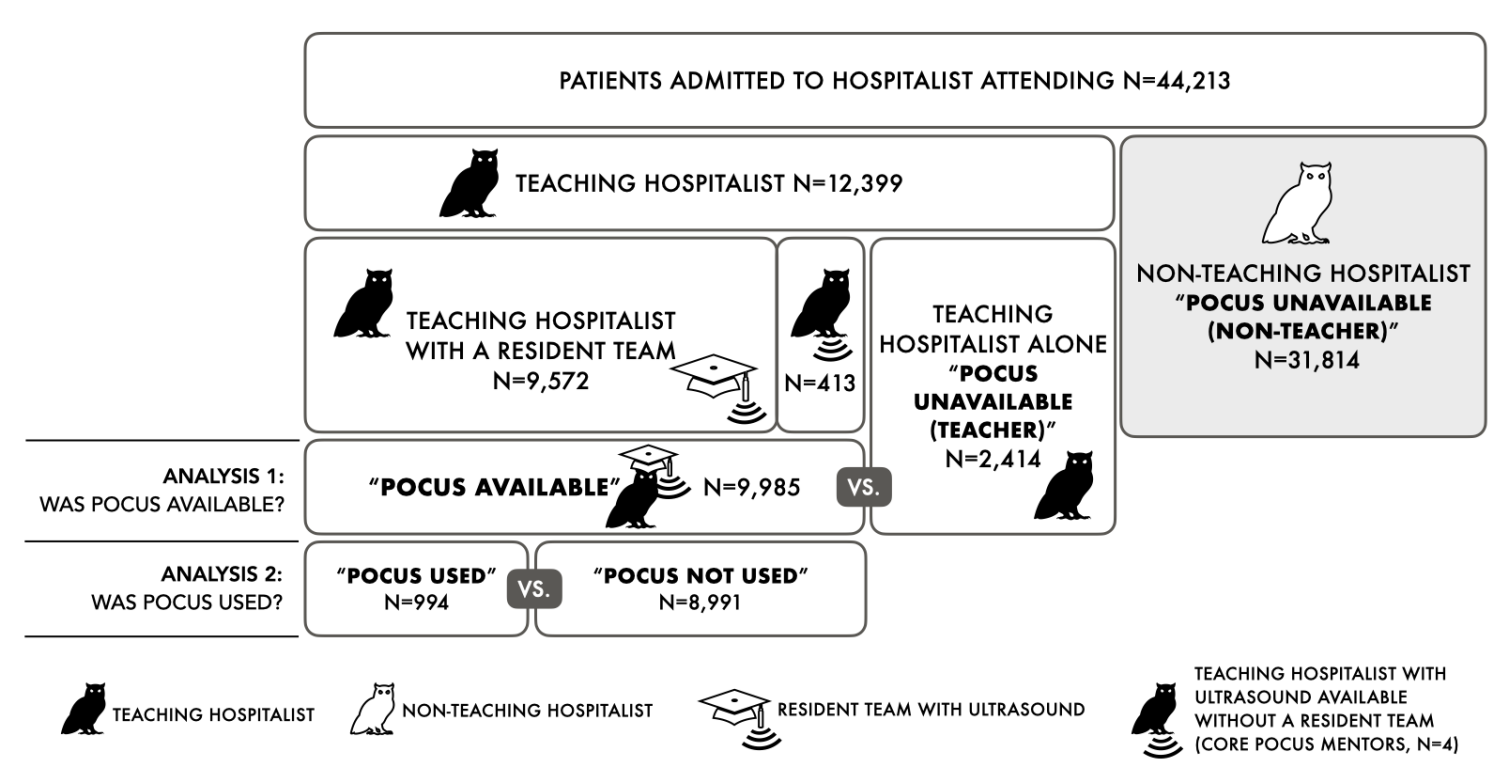
Overall and subgroup patient populations during the study time period with icons denoting the hospitalist type and presence of internal medicine resident teams with POCUS available for patient care. Subgroups analyzed in analyses 1 and 2 (see Methods: Statistical Analysis section) are also depicted in the figure. All n values represent number of patients in the subgroup. POCUS: point of care ultrasound.

Consecutive patients, age >18 years, admitted to IM were sequentially assigned to a hospitalist attending (Figure 1; N=44,213 patients, N=67 hospitalists). The hospitalist was either part of the resident faculty (“teaching hospitalist”; n=22) or not (“non-teaching hospitalist”, n=45). Among patients assigned to a teaching hospitalist, concomitant assignment to a resident team was based on whether the teaching hospitalist was 1) actively attending with a resident team that week (versus working independent of a resident team), and 2) if actively attending whether their resident team was available for admissions. So within a given attending week, a teaching hospitalist routinely cared for patients both with a resident team and without. Additionally, within a given month, a teaching hospitalist had weeks where they were not attending and cared for an entire census of patients without resident team involvement. All teaching hospitalists were full time, and spent roughly the same amount of time as each other in their roles as active resident team attending and independent hospitalist. Pertinent to this study, only patients cared for by a teaching hospitalist with a resident team had POCUS available and were classified as “POCUS available”. In addition, four teaching hospitalists who were core ultrasound mentors had constant POCUS access regardless of resident involvement, so patients cared for by these hospitalists were included in the “POCUS available” group even without resident team involvement. Patients assigned to all other teaching hospitalists without resident team involvement, and those assigned to non-teaching hospitalists were classified as “POCUS unavailable”. Patients with “POCUS unavailable” were further classified into subgroups of “POCUS unavailable (teacher)” and “POCUS unavailable (non-teacher)”, respectively. 

Among patients in the “POCUS available” group (Figure 1), those who received at least one POCUS exam during their hospital stay were classified as “POCUS used” while those that did not undergo a recorded exam were classified as “POCUS not used”.

### Primary and Secondary Outcomes

The study’s co-primary outcomes were difference in total and per-day hospital costs between patients cared for with or without POCUS available. Secondary outcomes included the difference in the major cost subcomponents and key hospitalization metrics of LOS, formal imaging volumes and costs, patient satisfaction, 30-day readmission, and ED visits between patients cared for with vs. without POCUS available (analysis 1 below) and also between patients cared for with POCUS used vs. POCUS available but not used (analysis 2 below). Secondary outcomes also included difference in total and per-day hospital costs (primary outcome) but for the subgroups of POCUS used vs. POCUS available but not used. All study outcomes were pre-specified.

### Data Sources and Measures

**Figure 2:**
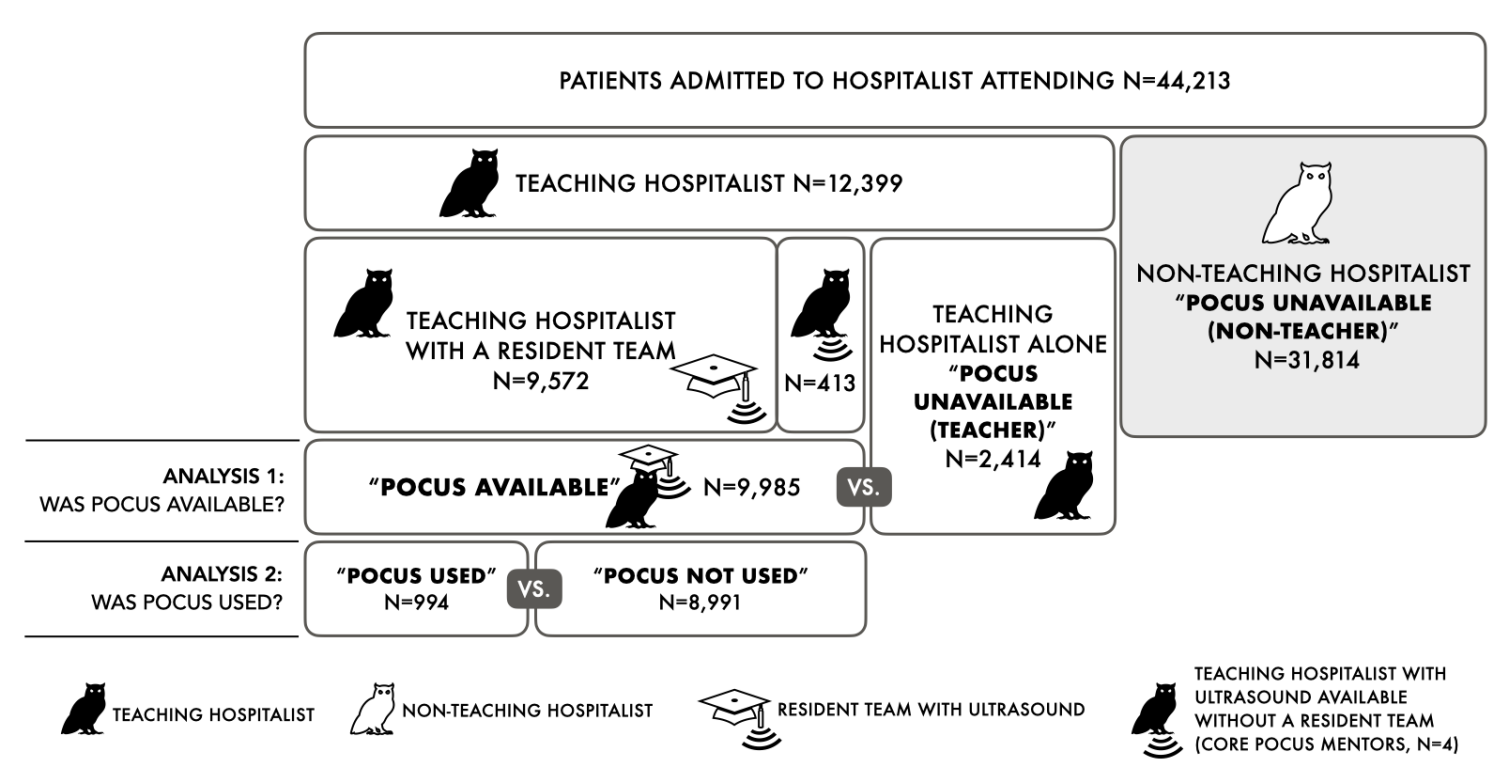
8,110 total body areas examined within 5,353 POCUS exams performed on 994 patients. HEENT: head, eyes, ears, nose, throat; MSKL: musculoskeletal.

Data were extracted from our POCUS smartphone application (all POCUS-related variables), the hospital’s electronic health record (EHR) (patient demographics, admission diagnosis, formal imaging studies, utilization and outcomes, care team identifiers), and the hospital billing database (costs). Care team identifiers determined involvement of hospitalist type (teaching, non-teaching) and resident team (yes, no) for each admission. In addition to POCUS exams, other imaging utilization variables included chest X-ray (CXR), chest computed tomography (CT), echocardiogram, and radiology-based ultrasound. Patient EHR data were used to compute a severity of illness (SOI) index using an established algorithm 15, 16. Outcomes included imaging utilization, hospital length-of-stay (LOS), Hospital Consumer Assessment of Healthcare Providers and Systems (HCAHPS) survey scores 17, and total hospitalization and radiology costs. Total hospitalization costs included all fixed and variable costs charged for these subcategories: drug supply, lab, radiology, room, operating room, respiratory care, therapy, other, and unclassified. Prespecified active hospital diagnosis subgroups included pneumonia, congestive heart failure (CHF), and acute kidney injury (AKI). These 3 subgroups were chosen a priori in light of our predominant cardiac, pulmonary, and bladder/kidney POCUS use patterns (Figure 2) and the potential of POCUS to impact those 3 patient populations differently.

### Statistical Analysis

Prior to outcome comparisons based on POCUS availability or use, two potential confounders associated with the availability of POCUS were assessed: impact of resident involvement in a patient’s care irrespective of their tie to POCUS availability/use (the “resident confounder”), and the impact of a hospitalist who is part of the teaching faculty vs. a non-teaching hospitalist caring for a patient irrespective of their tie to the presence of a resident team (the “teacher confounder”). The resident confounder was accounted for by matching patients on assignment to a resident team (yes, no) and subsequent gamma distribution or negative binomial regression to adjust for other variables. Similarly, the teacher confounder was accounted for by matching patients on whether care was provided by a teaching hospitalist (yes, no). The teacher confounder would have only needed to be accounted for in analyses involving non-teaching hospitalist patients (Figure 1, shaded box) as the comparison group—which we chose not to use for the analyses below (see Discussion section for rationale).

Analysis 1a (Figure 1) compared outcomes for patients with POCUS available (teaching hospitalist with a resident team, or one of the four core POCUS mentors alone) vs. patients cared for by the same teaching hospitalists with POCUS unavailable using multivariable analyses. This eliminated the need to account for the significant and complex “teacher confounder” introduced if using the “POCUS unavailable, non-teacher” group of patients and allowed attendings to serve as their own comparator. However, the “resident confounder” was present and needed to be accounted for in analysis 1a and 1b (below). Multivariable outcome analyses including patient age, sex, admission diagnosis grouping, SOI index 15, 16, and the “resident confounder” were performed. After adjusting for variables, least square means were established with gamma distribution for cost analyses, negative binomial regression for LOS analyses, Poisson distribution for imaging counts, and ordered logistic regression for HCAHPS scores. The same (1a) multivariable analysis was also performed by active diagnosis subgroup (CHF, pneumonia, AKI).

Analysis 1b compared the primary outcomes of total and per-day hospital costs for the same groups as 1a but instead used a 1:1 propensity score matching (PSM) analysis and now included LOS in the matching criteria: age, sex, race, admission diagnosis category, SOI, and LOS.

Analysis 2 compared patients within the POCUS available group that actually received at least one POCUS exam, of at least one body area, at any point during hospitalization vs. patients within the POCUS available group for whom the team elected to never utilize POCUS. A 1:4 PSM analysis was performed including the same covariates as analysis 1b.

Unless otherwise stated, all summary statistics are predicted means from the regression models accompanied by their 95% confidence interval that estimate the degree of precision in the predicted means. 

## Results

Patients (N=12,399) were consecutively admitted to the group of 22 teaching hospitalists during the study period (Table 1) with POCUS available for 80% of admissions (n=9,985). Active hospital diagnoses of pneumonia, CHF, and AKI were present in 11%, 22%, and 22% of patients, respectively. Most patients had a SOI index of 2 (37%) or 3 (38%) 15, 16.

**Table 1:** Patient characteristics

-	**Analysis 1a - MVR**			**Analysis 1b - 1:1 PSM**			**Analysis 2 - 1:4 PSM**		
Characteristic	POCUS available	POCUS not available	P	POCUS available	POCUS not available	P	POCUS used	POCUS not used	P
n	9,985	2,414	--	2,402	2,402	--	869	3,476	--
Age, mean (sd)	65.4 (18.5)	61.2 (17.8)	< 0.001	61.41 (18.47)	61.23 (18.54)	0.73	67.5 (17.0)	67.7 (17.1)	0.76
Sex, female, n (%)	5,260 (52.7)	1,235 (51.2)	0.18	1,221 (50.8)	1,228 (51.1)	0.84	416 (47.9)	1,743 (50.1)	0.23
Race, white, n (%)	8,077 (80.9)	2,076 (86.0)	<0.001	2,080 (86.6)	2,066 (86.0)	0.56	702 (80.8)	2,840 (81.7)	0.53
In-hospital mortality, n (%)	223 (2.2)	115 (4.8)	<0.001	95 (4.0)	114 (4.8)	0.18	33 (3.8)	109 (3.1)	0.33
Diagnosis subgroups, n (%)	--	--	--	--	--	--	--	--	--
Pneumonia	1,055 (10.6)	277 (11.5)	0.20	292 (12.2)	276 (11.5)	0.48	171 (19.7)	609 (17.5)	0.14
Congestive heart failure	2,215 (22.2)	504 (20.9)	0.16	474 (19.7)	498 (20.7)	0.39	310 (35.7)	1,306 (37.6)	0.30
Acute kidney injury	2,204 (22.1)	562 (23.3)	0.20	590 (24.6)	557 (23.2)	0.26	255 (29.3)	1,027 (29.6)	0.91
Severity of illness, n (%)	--	--	<0.001	--	--	0.90	--	--	0.96
1	1,320 (13.3)	341 (14.2)	N/D	336 (14.0)	341 (14.2)	N/D	48 (5.5)	203 (5.8)	N/D
2	3,816 (38.4)	769 (32.0)	N/D	769 (32.0)	769 (32.0)	N/D	230 (26.5)	928 (26.7)	N/D
3	3,915 (39.4)	814 (33.9)	N/D	837 (34.9)	814 (33.9)	N/D	457 (52.6)	1,831 (52.7)	N/D
4	896 (9.0)	478 (19.9)	N/D	460 (19.2)	478 (19.9)	N/D	134 (15.4)	514 (14.8)	N/D
Diagnosis subgroups represent prespecified diagnoses that were active during a given patient’s hospitalization. MVR: multivariable regression; N/D: not determined; POCUS: point-of-care ultrasound; PSM: propensity score matched									

For the 9,985 hospitalizations POCUS was available, it was used for 994 patients who underwent 5,353 exams covering 8,110 areas (Figure 2). Pulmonary (37%), cardiac (36%), and abdominal (23%) areas were most frequently performed. Exams combined pulmonary and cardiac areas 56% of the time.

### Analysis 1: POCUS available vs. not available

All values in analysis 1 and 2 are means unless otherwise specified. Their associated 95%CI can be found in the referenced tables 2 and 3. 

**Table 2:** Patient and system outcomes – analysis 1a and 1b

-	**Analysis 1a - Multivariable Regression Analysis**			
Outcome	POCUS available	POCUS not available	Difference	P
n	9,985	2,414	--	--
Length of stay (days)	5.77 (5.63 - 5.91)	6.08 (5.66 - 6.51)	-0.31	0.14
Total hospitalization cost ($)	17,474 (16,397 - 18,010)	21,803 (20,212 - 23,393)	-4,329	<0.001
Hospitalization cost per day ($)	2,805.88 (2,761.63 - 2,850.13)	3,557.53 (3,426.33 - 3,688.72)	-751.65	<0.001
Total hospitalization radiology cost ($)	705.41 (680.57 - 730.25)	829.12 (755.38 - 902.67)	-123.71	<0.001
Radiology cost per day ($)	163.11 (155.59 - 170.62)	198.53 (176.23 - 220.84)	-35.42	<0.001
Chest X-Ray total/hospitalization	1.31 (1.26 - 1.37)	1.55 (1.38 - 1.72)	-0.24	0.01
Chest X-Ray per day	0.22 (0.22 - 0.23)	0.27 (0.24 - 0.29)	-0.04	0.09
Chest CT total/hospital stay	0.22 (0.21 - 0.23)	0.15 (0.11 - 0.19)	0.07	0.001
Chest CT per day	0.04 (0.04 - 0.05)	0.03 (0.02 - 0.04)	0.01	0.26
Echocardiogram total/hospital stay	1.21 (1.17 - 1.24)	1.36 (1.26 - 1.46)	-0.15	0.19
Echocardiogram per day	0.38 (0.37 - 0.40)	0.41 (0.37 - 0.45)	-0.03	0.62
Ultrasound total/hospital stay	0.35 (0.33 - 0.37)	0.30 (0.24 - 0.35)	0.05	0.04
Ultrasound per day	0.08 (0.07 - 0.08)	0.07 (0.05 - 0.09)	0.01	0.73
-	**Analysis 1b - 1:1 Propensity Score Matched**			
n	2,402	2,402	--	--
Total hospitalization cost ($)	19,905 (17,508 - 22,301)	21,490 (19,093 - 23,888)	-1,585	0.01
Hospitalization cost per day ($)	2,776.46 (2,606.55 - 2,946.38)	3,441.35 (3,271.39 - 3,611.32)	-664.89	<0.001
Analysis 1a and 1b compare outcomes between patients cared for by hospitalists with POCUS available but not necessarily used, and those cared for without POCUS available using multivariable regression (length of stay as dependent variable) and 1:1 propensity score matched (length of stay as covariate) analyses. All values are mean (95% CI) unless otherwise noted. POCUS: point-of-care ultrasound; PSM: propensity score matched				

**Table 3:** Patient and system outcomes – analysis 2

-	**Analysis 2 - 1:4 Propensity Score Matched**			
Outcome	POCUS used	POCUS not used	Difference	P
n	869	3,476	--	--
Total hospitalization cost ($)	15,082 (14,439 - 15,725)	15,746 (15,364 - 16,127)	-664	<0.001
Hospitalization cost per day ($)	2,685 (2,592 - 2,778)	2,753 (2,698 - 2,808)	-68	0.04
Total hospitalization radiology cost ($)	642 (590 - 694)	630 (599 - 661)	12	0.23
Radiology cost per day ($)	146 (132 - 161)	154 (145 - 163)	-7	0.17
Chest X-Ray total/hospitalization	1.64 (1.51 - 1.77)	1.16 (1.08 - 1.24)	0.48	< 0.001
Chest X-Ray per day	0.31 (0.28 - 0.33)	0.25 (0.23 - 0.26)	0.06	0.001
Chest CT total/hospital stay	0.30 (0.26 - 0.33)	0.20 (0.18 - 0.22)	0.10	< 0.001
Chest CT per day	0.06 (0.05 - 0.07)	0.05 (0.04 - 0.05)	0.01	0.07
Echocardiogram total/hospital stay	0.43 (0.38 - 0.48)	0.34 (0.31 - 0.37)	0.10	< 0.001
Echocardiogram per day	0.09 (0.08 - 0.11)	0.07 (0.07 - 0.08)	0.02	0.10
Ultrasound total/hospital stay	1.30 (1.23 - 1.37)	1.17 (1.12 - 1.22)	0.13	0.05
Ultrasound per day	0.33 (0.30 - 0.35)	0.37 (0.35 - 0.38)	-0.04	0.86
Analysis 2 includes patients cared for by hospitalists with POCUS available for use and compares outcomes between patients where POCUS was chosen to be used and patients where POCUS was available but not chosen to be used using 1:4 propensity score matched (length of stay as covariate) analyses. All values are mean (95% CI) unless otherwise noted. POCUS: point-of-care ultrasound; PSM: propensity score matched				

Analysis 1a, multivariable analyses comparing hospitalizations where POCUS was available vs. not available (Figure 1), showed a LOS of 5.77 vs. 6.08 days (P=0.14), respectively (Table 2). Total and per-day hospitalization costs were significantly lower in the POCUS available group ($-4,329, P<0.001; $-751, P<0.001, respectively) as were total and per-day radiology costs ($-124, P<0.001; $-35, P<0.001, respectively). Radiology cost reduction among POCUS available patients was driven by a decrease in total CXRs (1.31 vs. 1.55, P=0.005) and tempered by an increase in total chest CT (0.22 vs. 0.15, P=0.001) and total formal ultrasounds (0.35 vs. 0.30, P=0.04).

When imaging outcomes were analyzed by diagnosis subgroups, total CXR use was reduced by 36% in CHF patients with POCUS available (1.63 vs. 2.56, P<0.001); however, formal echocardiograms were not significantly reduced (1.28 vs. 1.59, P=0.145). The AKI subgroup had 42% lower total CXR use when POCUS was available (1.62 vs. 2.79, P=0.001). Patients with pneumonia accounted for most of the chest CT increase seen in the POCUS available cohort (0.51 vs. 0.12, P<0.001) (Appendix 2: Admission subgroup analysis).

Analysis 1b re-analyzed the primary outcome of hospital costs between POCUS available and not available cohorts using a 1:1 PSM analysis including LOS as a covariate (unlike 1a where LOS was a dependent variable) and demonstrated total hospital and per-day costs remained significantly lower with POCUS available ($19,905 vs. $21,490, P=0.008; $2,776 vs. $3,441, P<0.001). 

30-day hospital readmission, ED visits, and HCAHPS question scores (Appendix 3: HCAHPS survey) targeting physician courtesy/respect, listening, explaining understandably, as well as overall hospitalization ratings did not differ significantly between groups.

### Analysis 2: POCUS available and used vs. available but not used

In this cohort design, providers with POCUS available chose to use it on some patients but not others for unknown reasons that may impact outcomes. Therefore, comparison of these two groups does not directly assess the value of POCUS and introduces potential confounding. This 1:4 PSM analysis (including LOS as a covariate because of its non-linear impact on variable costs and potential influence on decisions to use ultrasound) was chosen to compare the two subgroups of patients to minimize unaccounted for confounders (Table 1). It showed a significant reduction in total and per-day hospitalization costs, a reduction in total and per-day CXRs, as well as a small increase in chest CT and formal echocardiograms in the subgroup where POCUS was chosen for use (Table 3).

## DISCUSSION

Through multiple analytic lenses, POCUS availability and selected use by IM hospitalist teaching teams was significantly, and meaningfully associated with reduced hospital costs and CXR use with a slight increase in chest CT. The “POCUS available vs. not available” analysis (1a) probably best reflects a real-world, system-level approach to a POCUS implementation’s per-day cost savings in which devices are available for provider use based on clinical circumstance, but where POCUS isn’t mandated for a group of patients. Total hospital cost savings in this primary analysis is certainly driven partly by lower LOS which, importantly, may be in part due to POCUS but is at risk for confounding overestimating its benefit. However, the 1:1 PSM re-analysis (1b) containing LOS as a covariate maintained significant differences in both total and per-day costs increasing the probability that POCUS was impacting outcomes and likely underestimating its benefit through some reduction in LOS. In every environment there will be patients who would have benefited from POCUS but did not receive it, and patients who did not benefit or even had additional cost or LOS because of an incidental finding. This study design and analysis 1 accounts for those aspects of a real-world POCUS implementation better than a randomized trial might.

The primary cost endpoints were further evaluated between the “POCUS available” subgroups that had “POCUS used” vs. those that “could have but didn’t” (Analysis 2) and included LOS as a covariate instead of a dependent variable. The more modest cost savings seen in this analysis likely reflects the non-linear relationship of LOS (covariate in this analysis, dependent variable in analysis 1a) vs. variable hospital costs, significantly lower total and daily hospitalization costs in the “POCUS not used” cohort, and the presence of other cost or LOS-increasing confounders triggering POCUS use by providers such as higher illness burden (not captured by SOI index), lack of timely improvement prompting POCUS use, etc. Despite these potential cost-increasing selection biases for POCUS use, savings associated with POCUS use were maintained. 

CXR reduction is a well-known POCUS benefit, has been shown in ICU and pediatric studies 18, 19, and is consistent with our results. The small but identifiable increase in chest CT is also congruent with our clinical experience. Rarely is CXR additive to a thorough pulmonary POCUS exam, so a CT is typically the appropriate next test. The diagnostic testing sequence of POCUS + CT is overall less radiation and cost compared to CXR + CT with better test characteristics 20. The minimal reduction in CXR seen in the pneumonia “POCUS available” subgroup may seem counterintuitive; however, these CXRs are usually obtained in the ED or outpatient setting prior to hospital admissions and rarely repeated. The largest reduction in CXR use was seen in patients with CHF and AKI active during their hospitalization where volume status and ongoing diuresis decisions may traditionally prompt a CXR—information more accurately gathered with a cardiopulmonary POCUS exam 20, 21, 22, 23, 24.

There is a paucity of POCUS cost effectiveness research with virtually none within IM. Cost’s main determinant, LOS, has more, but still very limited data. Recently, Matthews et al. presented a $743 reduction in patient cost for each POCUS exam performed by a hospitalist (n=50) 25. An Italian cost modeling study in 2015 demonstrated the equivalence of ~$128 cost savings per hospital exam and a break-even point for a program of 734 exams 26. An Australian trial in hospitalized patients with cardiopulmonary symptoms receiving cardiac, lung, and vascular POCUS at admission (n=124) vs. no POCUS demonstrated no significant difference in LOS (113 vs. 125 hr), 30-day readmission (16% vs. 12%), or total costs (~$6,030 vs. ~$6,079, respectively) 21. Mozzini et al. found a 1 day reduction in LOS in hospitalized CHF patients receiving multiple lung ultrasound exams vs. CXR guiding diuresis 27. Lucas et al. did not identify a significant decrease in LOS for hospital patients receiving hand-carried echocardiography (n=226), but did identify a significant 17% reduction in a subgroup of patients with CHF 10. In contrast to these previous studies, our study includes the most patients of any published study, with any admission diagnosis, a large amount (23%) of abdominal POCUS, and multiple patients receiving repeat exams compared to single admission exams. An important efficiency gained from IM POCUS may be in following changes with treatment or use when patients are not responding as expected after admission—not captured in studies with a single admission POCUS.

Our results represent a diverse, quaternary-care, patient population at a large urban teaching hospital with a robust POCUS training program, but should be cautiously extrapolated to other settings. We show outcomes from the early years of our program because it availed the unique setting of attendings functioning as their own “control”. Secondly, the outcomes of a program after 8-10 years of maturation may be less meaningful for programs attempting to justify initial POCUS implementation. As a program matures, provider skill and recognition of normal variation increases, POCUS as a language of communication becomes fluent between providers, and system efficiencies likely increase. 

A decision was made to compare patients cared for with and without POCUS (n=12,399) among the same group of teaching hospitalists (comparing teaching hospitalists to themselves in the 4 study cohorts and 2 analyses) rather than the alternative of comparing patients cared for with POCUS by teaching hospitalists to the much larger cohort of patients (n=31,814) cared for without ultrasound by non-teaching hospitalists. The subgroup of teaching hospitalists invited to that teaching role differ on average from our non-teaching hospitalists in complex ways, some of which (experience, efficiency, personality, test ordering patterns, etc.) have potential to impact chosen study outcomes in ways unrelated to POCUS. The use of a regression analysis that attempted to adjust for this complex set of characteristics between 2 different groups of hospitalists (using the “teacher confounder”) with the goal of ultimately isolating POCUS as the variable impacting study outcomes such as LOS, radiology test ordering, patient satisfaction, etc. was methodologically weak. We believe the methodologic decision to compare teaching hospitalists to themselves, despite decreasing the overall number of patients in the study, results in a more valid assessment of POCUS’ impact on our study outcomes.

Two audits during our program’s evolution demonstrated recording rates for POCUS exams were 87-91%; thus, there were likely patients misassigned to the analysis 2 “was not used” group (~10%)—theoretically underestimating POCUS’ benefit in analysis 2 but not impacting analysis 1. Proportion of exams changing management was not tracked; however, it likely resembles that of most POCUS programs during the early years of development. Program implementation costs (equipment, training, consumables, physician time) were not analyzed as this is institution-specific and rapidly changing. We did not compile all hospital diagnoses in this study but expect over the 4-year study period that the diagnosis mix would be reasonably balanced between the POCUS available and not available cohorts due to the allocation algorithm of patient admissions to hospitalists and resident teams. Thus, the actual diagnosis mix within a cohort should not impact the primary and secondary outcomes assessing difference between the POCUS available and not available cohorts. Additional savings from LOS reduction when POCUS can replace the need to wait for formal imaging studies may be seen in other settings, but contributed minimally in our setting where imaging was readily available 26. Similarly, POCUS for procedural guidance can decrease LOS when delays in radiology-based scheduling exist, reduce unnecessary procedures, and reduce complications associated with landmark-based procedures 28, 29. These potential benefits for other groups were not part of our analysis as all patients had readily accessible ultrasound-guided bedside procedures through our procedure team. Potential unaccounted for confounders contributing to over- or under-estimation of POCUS’ benefit are possible in the PSM analyses. Finally, this study did not evaluate potential harm from POCUS specifically, but there was no difference in 30-day readmission or ED visits for patients in the POCUS group, and most other potential harms would otherwise be reflected in additional LOS and cost.

Acknowledging these limitations, this study builds on currently available anecdotal experience and smaller studies demonstrating the impact of POCUS within IM. Actual system and patient benefits attributable to IM POCUS alone likely resides somewhere between the two lenses it has been examined through in this large prospective cohort study. However, like for many other tools physicians utilize daily with great benefit, we are unlikely to see a randomized trial that is capable of truly isolating the causal impact of POCUS on hospital metrics for the reasons previously discussed and limitations of randomized trials.

## Ethics approval and consent to participate

Patient consent obtained for research participation; ethics approval waived. IRB approval Reference 201306503

## Competing Interests

TR, LS, KH, CS, LB, RM all declare no potential conflicts of interest related to the content of this manuscript. DT serves on the POCUS Task Force for the Society of Hospital Medicine and American College of Physicians (ACP), is the co-director for the ACP Fundamentals of POCUS course with honoraria, has previously served on the medical advisory board for EchoNous, Inc. and currently holds stock and stock options.

## Funding

The Abbott Northwestern Hospital Foundation provided funding for study design consultation and statistical analysis. The source had no role in conducting the study, analysis, or manuscript prep/review.

## Author Contributions

All authors have contributed significantly to and approved the final manuscript. DT, TR, RM contributed to study design and writing & revision of the manuscript. KH contributed to study design, data analysis. CS, LB contributed to study design, data analysis, and writing & revision of the manuscript. LS contributed significantly to writing & revision of the manuscript. 

## Supplementary Material

 Supplementary Appendix Supplementary Appendix A-C
