# Supplementary material for: Association of Internal Medicine Point of Care Ultrasound (POCUS) with Length of Stay, Hospitalization Costs, and Formal Imaging: a Prospective Cohort Study
Source: POCUS J. 2023 Nov 27;8(2):184–92. doi: 10.24908/pocus.v8i2.16791 (PMC10721304; doi:10.24908/pocus.v8i2.16791)
Supplement: Supplementary Appendix [file pocusj-08-16791-s001.pdf]

Supplemental Digital Content 1: “IMBUS Certification Areas & Items”

Exam Area	Exam Item	Certification Cutpoint	Core Element
**ABDOMINAL**			
	Liver Size	8	x
	Hepatomegaly	5	x
	Morrison’s	8	x
	Liver Mass	3	
	Liver Steatosis	3	
	Ascites	5	x
	Spleen Size	8	x
	Splenomegaly	5	x
	Splenorenal	8	x
	Kidney – Normal	12	x
	Kidney – Hydro	8	x
	Kidney - CKD	5	
	Kidney - Mass	3	
	Kidney - Cyst	8	x
	Bladder Volume	8	x
	Prostate Normal	6	
	Prostate Enlarged	6	
	Uterus/Ovaries Normal	6	
	Uterus/Ovaries Abnormal	3	
	GB - Normal	20	x
	GB - Gallstones	8	x
	GB – Cholecystitis	6	
	GB - CBD dilated	6	
	GB - CBD normal	15	
	Hernia	5	
	SBO	5	
	Ileus	5	
**CARDIAC**			
	3-4 Views	50	x
	IVC Assessment	15	x
	Normal Function	15	x
	Hyperdynamic LV	8	x
	Severe Hypo LV	10	x
	Mild/Mod Hypo LV	18	x
	Segmental WMA	10	
	Pericardial Effusion	8	x
	Tamponade	5	
	RV Enlargement	10	x
	Left Pleural Effusion-Heart	5	x
	Right Pleural Effusion-Heart	3	
	Pleural and PCE	8	
	LV Wall Thickening	8	x
	Mitral Regurg	15	x
	Mitral Valve SAM	5	
	Mitral Stenosis/MAC	3	
	Tricuspid Regurg	15	x
	Aortic Regurg	15	x
	Aortic Stenosis	15	x
	Ao Root Dilation	10	
	Diastolic Normal	15	
	Diastolic Dysfunction	15	
**HEAD & NECK**			
	Sinus_NL	10	x
	Sinus_ABNL	3	x
	Thyroid – Normal	15	
	Thyroid – Abnormal	10	
	Ocular – Normal	10	
	Ocular – Abnormal	5	
	Optic Nerve Sheath	6	
**MUSCULOSKELETAL**			
	Knee Effusion	5	x
	Rib Fracture	4	
	Fracture (not rib)	5	
	Trochanteric Bursa	4	
	Baker's Cyst	4	
	Gout/Pseudo	4	
	Ganglion Cyst	3	
	Bursitis	5	
	Synovitis	5	
	Tendonitis/osis	5	
	Tear-Lig/Tendon	5	
	Tear-Muscle	5	
**PULMONARY**			
	Zones 1-4	15	x
	Zones 5-6	15	x
	Lung Sliding	10	x
	Pleural Effusion	10	x
	Interstitial Pattern	15	x
	Atelectasis	8	x
	Pneumonia/Consol	15	x
	Subpleural Consolidation	15	x
	Pneumothorax	5	x
**SOFT TISSUE**			
	Cellulitis	5	x
	Abscess	5	x
	Lymph N - Normal	8	x
	Lymph N - Abnormal	5	x
	Lymph N - Malignant	8	
	Cyst	8	
	Lipoma	8	
**VASCULAR**			
	IJ/CVP - Normal	8	
	IJ/CVP - Elevated	8	
	DVT Screen - Normal	25	
	DVT Screen - Abnormal	5	
	AAA Screen – Normal	25	
	AAA Screen - Abnormal	8	
	Carotid - Normal	15	
	Carotid - Plaque	8	
	Carotid - FlowTime	10	

Listing of exam items by area that are tracked within the IMBUS program for certification. Minimum number of exams required to be eligible for certification assessment within each exam item is listed. Achievement of minimum quantities is requisite for but not the only aspect required for certification in the item. Core elements noted are required for residency graduation.

Appendix 2: Total and per-day imaging counts for patients in the "POCUS available" and "POCUS not available" admission diagnosis subgroups

Outcome	POCUS available	POCUS not available	Difference	P
Pneumonia subgroup (n)	1055	277	n/a	n/a
Chest X-ray total	2.194	2.685	-0.491	0.784
Chest X-ray per day	0.377	0.442	-0.065	0.539
CT chest total	0.511	0.117	0.394	<0.001
CT chest per day	0.105	0.068	0.037	0.377
Ultrasound total	0.408	0.426	-0.018	0.628
Ultrasound per day	0.041	0.065	-0.024	0.530
Echocardiogram total	1.275	1.551	-0.276	0.388
Echocardiogram per day	0.314	0.337	-0.023	0.824
CHF subgroup (n)	2215	504	n/a	n/a
Chest X-ray total	1.634	2.556	-0.922	<0.001
Chest X-ray per day	0.310	0.385	-0.075	0.259
CT chest total	0.226	0.119	0.107	0.049
CT chest per day	0.041	0.004	0.037	0.106
Ultrasound total	0.408	0.517	-0.109	0.204
Ultrasound per day	0.073	0.070	0.003	0.945
Echocardiogram total	1.279	1.591	-0.312	0.145
Echocardiogram per day	0.306	0.333	-0.027	0.792
Acute kidney injury subgroup (n)	2204	562	n/a	n/a
Chest X-ray total	1.623	2.793	-1.170	0.001
Chest X-ray per day	0.243	0.302	-0.059	0.314
CT chest total	0.191	0.187	0.004	0.621
CT chest per day	0.029	0.030	-0.001	0.950
Ultrasound total	0.566	0.392	0.174	0.022
Ultrasound per day	0.093	0.063	0.030	0.385
Echocardiogram total	1.342	1.499	-0.157	0.547
Echocardiogram per day	0.347	0.316	0.031	0.709

CHF=congestive heart failure; CT=computed tomography; POCUS=point-of-care ultrasound
All values are means unless otherwise noted

Appendix 3: Hospital Consumer Assessment of Healthcare Providers and Systems (HCAHPS) survey – selected questions and scores

Appendix Table: Mean score for selected HCAHPS questions among patients in the "POCUS available" and "POCUS not available" groups (see survey on next page for full questions)

Survey question # - topic	POCUS available	POCUS not available	Difference	P
5 - Courtesy and respect	3.89	3.81	0.08	0.35
6 - Listen carefully	3.73	3.78	-0.05	0.39
7 - Explain understandably	3.65	3.65	0	0.79
21 - Hospital rating overall	9.11	8.9	0.21	0.74
22 - Recommend hospital	3.8	3.72	0.08	0.37

POCUS=point-of-care ultrasound

SURVEY INSTRUCTIONS: You should only fill out this survey if you were the patient during the hospital stay named in the cover letter. Do not fill out this survey if you were not the patient. Answer all the questions by completely filling in the circle to the left of your answer. You are sometimes told to skip over some questions in this survey. When this happens you will see an arrow with a note that tells you what question to answer next, like this:

○ Yes
● No → **If No, Go to Question 1**

Please answer the questions in this survey about your stay at Regina Hospital. Do not include any other hospital stays in your answers.

Please use black or blue ink to fill in the circle completely. Example: ●

YOUR CARE FROM NURSES

1. During this hospital stay, how often did nurses treat you with courtesy and respect?
   - ○ Never
   - ○ Sometimes
   - ○ Usually
   - ○ Always

2. During this hospital stay, how often did nurses listen carefully to you?
   - ○ Never
   - ○ Sometimes
   - ○ Usually
   - ○ Always

3. During this hospital stay, how often did nurses explain things in a way you could understand?
   - ○ Never
   - ○ Sometimes
   - ○ Usually
   - ○ Always

4. During this hospital stay, after you pressed the call button, how often did you get help as soon as you wanted it?
   - ○ Never
   - ○ Sometimes
   - ○ Usually
   - ○ Always
   - ○ I never pressed the call button

YOUR CARE FROM DOCTORS

5. During this hospital stay, how often did doctors treat you with courtesy and respect?
   - ○ Never
   - ○ Sometimes
   - ○ Usually
   - ○ Always

6. During this hospital stay, how often did doctors listen carefully to you?
   - ○ Never
   - ○ Sometimes
   - ○ Usually
   - ○ Always

7. During this hospital stay, how often did doctors explain things in a way you could understand?
   - ○ Never
   - ○ Sometimes
   - ○ Usually
   - ○ Always

THE HOSPITAL ENVIRONMENT

8. During this hospital stay, how often were your room and bathroom kept clean?
   - ○ Never
   - ○ Sometimes
   - ○ Usually
   - ○ Always

9. During this hospital stay, how often was the area around your room quiet at night?
   - ○ Never
   - ○ Sometimes
   - ○ Usually
   - ○ Always

YOUR EXPERIENCES IN THIS HOSPITAL

10. During this hospital stay, did you need help from nurses or other hospital staff in getting to the bathroom or in using a bedpan?
    - ○ Yes
    - ○ No → **If No, Go to Question 12**

11. How often did you get help in getting to the bathroom or in using a bedpan as soon as you wanted?
    - ○ Never
    - ○ Sometimes
    - ○ Usually
    - ○ Always

12. During this hospital stay, did you need medicine for pain?
    - ○ Yes
    - ○ No → **If No, Go to Question 15**

13. During this hospital stay, how often was your pain well controlled?
    - ○ Never
    - ○ Sometimes
    - ○ Usually
    - ○ Always

14. During this hospital stay, how often did the hospital staff do everything they could to help you with your pain?
    - ○ Never
    - ○ Sometimes
    - ○ Usually
    - ○ Always

15. During this hospital stay, were you given any medicine that you had not taken before?
    - ○ Yes
    - ○ No → **If No, Go to Question 18**

16. Before giving you any new medicine, how often did hospital staff tell you what the medicine was for?
    - ○ Never
    - ○ Sometimes
    - ○ Usually
    - ○ Always

17. Before giving you any new medicine, how often did hospital staff describe possible side effects in a way you could understand?
    - ○ Never
    - ○ Sometimes
    - ○ Usually
    - ○ Always

Draft

continued...

WHEN YOU LEFT THE HOSPITAL

18. After you left the hospital, did you go directly to your own home, to someone else's home, or to another health facility?
    - O Own home
    - O Someone else's home
    - O Another health facility → **If Another, Go to Question 21**

19. During this hospital stay, did doctors, nurses or other hospital staff talk with you about whether you would have the help you needed when you left the hospital?
    - O Yes
    - O No

20. During this hospital stay, did you get information in writing about what symptoms or health problems to look out for after you left the hospital?
    - O Yes
    - O No

OVERALL RATING OF HOSPITAL

Please answer the following questions about your stay at the hospital named on the cover letter. Do not include any other hospital stays in your answers.

21. Using any number from 0 to 10, where 0 is the worst hospital possible and 10 is the best hospital possible, what number would you use to rate this hospital during your stay?
    - O 0 Worst hospital possible
    - O 1
    - O 2
    - O 3
    - O 4
    - O 5
    - O 6
    - O 7
    - O 8
    - O 9
    - O 10 Best hospital possible

22. Would you recommend this hospital to your friends and family?
    - O Definitely no
    - O Probably no
    - O Probably yes
    - O Definitely yes

UNDERSTANDING YOUR CARE WHEN YOU LEFT THE HOSPITAL

23. During this hospital stay, staff took my preferences and those of my family or caregiver into account in deciding what my health care needs would be when I left.
    - O Strongly disagree
    - O Disagree
    - O Agree
    - O Strongly agree

24. When I left the hospital, I had a good understanding of the things I was responsible for in managing my health.
    - O Strongly disagree
    - O Disagree
    - O Agree
    - O Strongly agree

25. When I left the hospital, I clearly understood the purpose for taking each of my medications.
    - O Strongly disagree
    - O Disagree
    - O Agree
    - O Strongly agree
    - O I was not given any medication when I left the hospital

ABOUT YOU

26. During this hospital stay, were you admitted to this hospital through the Emergency Room?
    - O Yes
    - O No

27. In general, how would you rate your overall health?
    - O Excellent
    - O Very good
    - O Good
    - O Fair
    - O Poor

28. In general, how would you rate your overall <u>mental or emotional health</u>?
    - O Excellent
    - O Very good
    - O Good
    - O Fair
    - O Poor

29. What is the highest grade or level of school that you have <u>completed</u>?
    - O 8th grade or less
    - O Some high school, but did not graduate
    - O High school graduate or GED
    - O Some college or 2-year degree
    - O 4-year college graduate
    - O More than 4-year college degree

30. Are you of Spanish, Hispanic or Latino origin or descent?
    - O No, not Spanish/Hispanic/Latino
    - O Yes, Puerto Rican
    - O Yes, Mexican, Mexican American, Chicano
    - O Yes, Cuban
    - O Yes, other Spanish/Hispanic/Latino

31. What is your race? Please choose one or more.
    - O White
    - O Black or African American
    - O Asian
    - O Native Hawaiian or other Pacific Islander
    - O American Indian or Alaska Native

32. What language do you <u>mainly</u> speak at home?
    - O English
    - O Spanish
    - O Chinese
    - O Russian
    - O Vietnamese
    - O Some other language (please print): ____________________

Patient's Name: ______________________ *(optional)* Telephone Number: ______________________ *(optional)*

THANK YOU. Please return the completed survey in the postage-paid envelope.

© 2004 PRESS GANEY ASSOCIATES, INC.,
All Rights Reserved
CL#3663-HZ0101U-00-03/14

Draft

PRESS GANEY™
